# Supplementary material for: Multi-dimensional scaling techniques unveiled gain1q&loss13q co-occurrence in Multiple Myeloma patients with specific genomic, transcriptional and adverse clinical features
Source: Nat Commun. 2024 Feb 20;15:1551. doi: 10.1038/s41467-024-45000-z (PMC10879136; doi:10.1038/s41467-024-45000-z)
Supplement: Supplementary file 1 — Supplementary Information [file 41467_2024_45000_MOESM1_ESM.pdf]

Multi-dimensional scaling techniques unveiled gain1q&loss13q co-occurrence in Multiple Myeloma patients with specific genomic, transcriptional and adverse clinical features.

Supplemental Appendix

Supplementary Figure S1

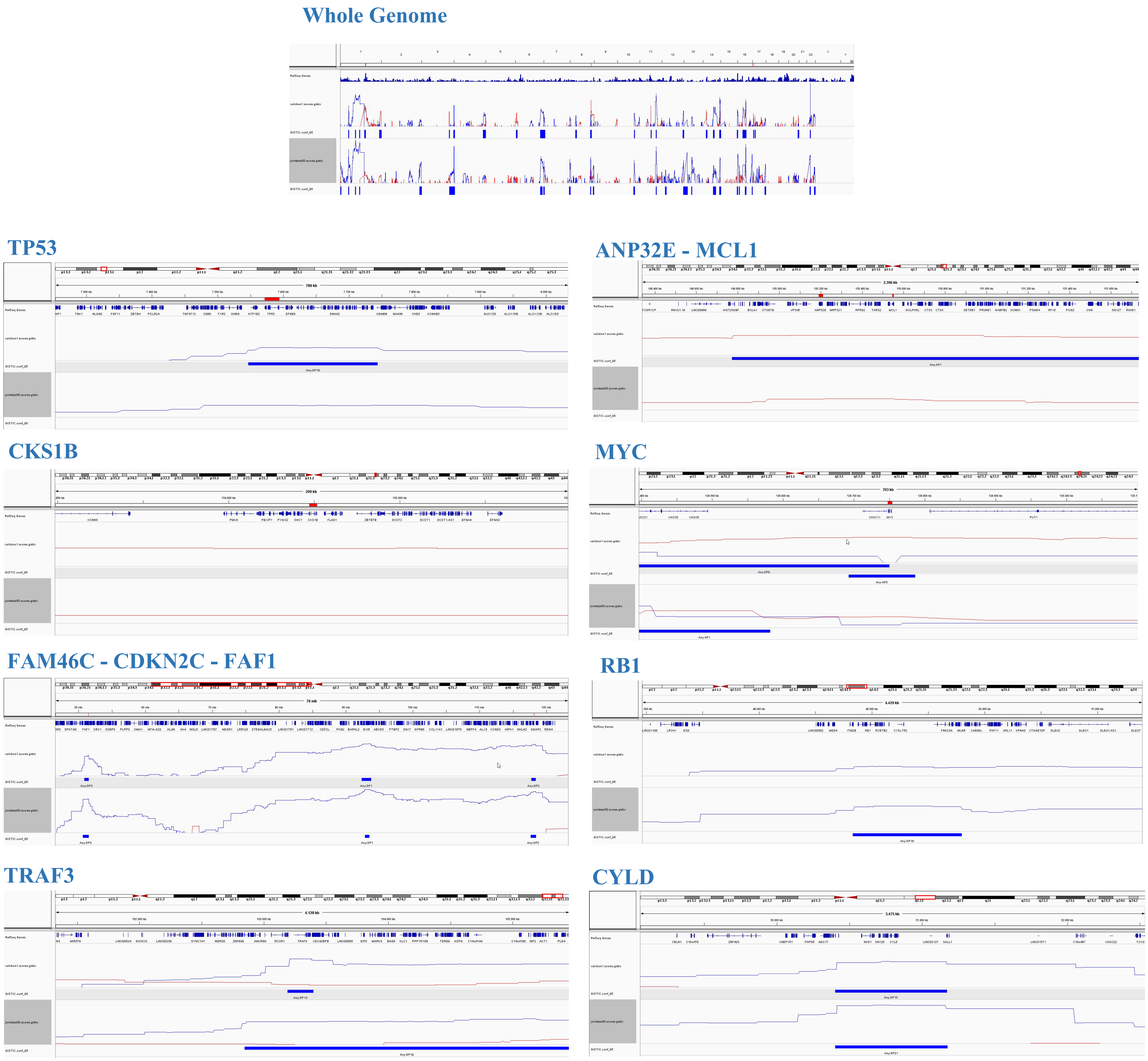

Supplementary Figure S1: IGV screenshots representing significative focal CNAs identified by GISTIC 2.0 algorithm in both MM-BO (upper tracks) and CoMMpass (lower tracks) datasets. Blue bars ("conf_95" files) highlight the significative regions; continuous blue and red lines ("scores.gistic" files) define the G-score along the chromosomes, for deletion and amplification, respectively.

Supplementary Figure S2

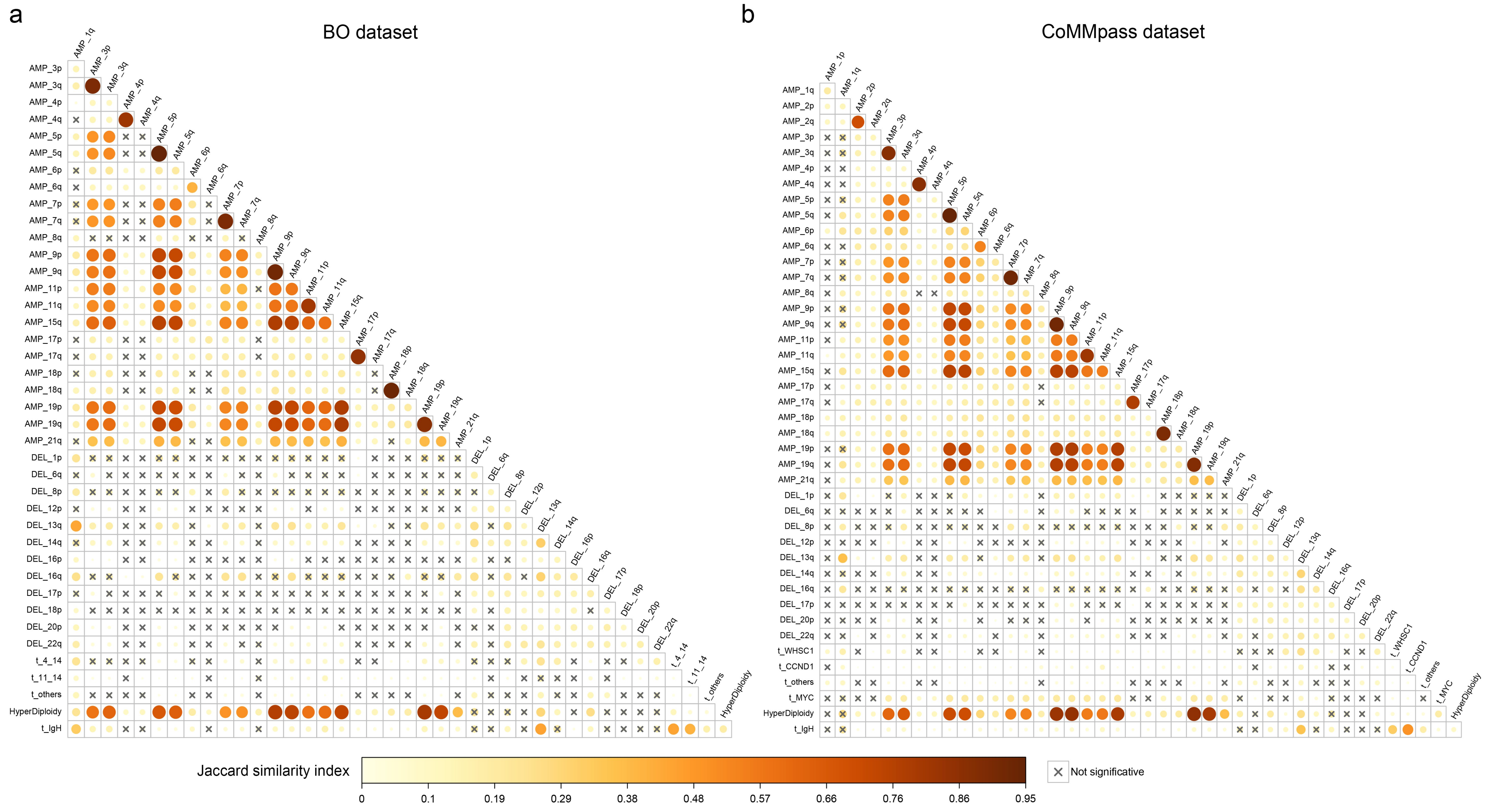

Supplementary Figure S2: Jaccard Similarity Matrix built to highlight pairwise similarities between genomic variables, treated as dichotomized data. All aberrations (with frequency>5%) in were included in the analysis for a) BO dataset, n=513 patients, and b) CoMMpass dataset, n = 752 patients. The heatmap resumed results coming from Jaccard similarity tests, performed for each couple of variables, as called when detected within a clonality > 50%. An X highlighted not-significant correlations (two-sided p>0.05). The approach to multiple testing correction is described in methods section.

Supplementary Figure S3

a

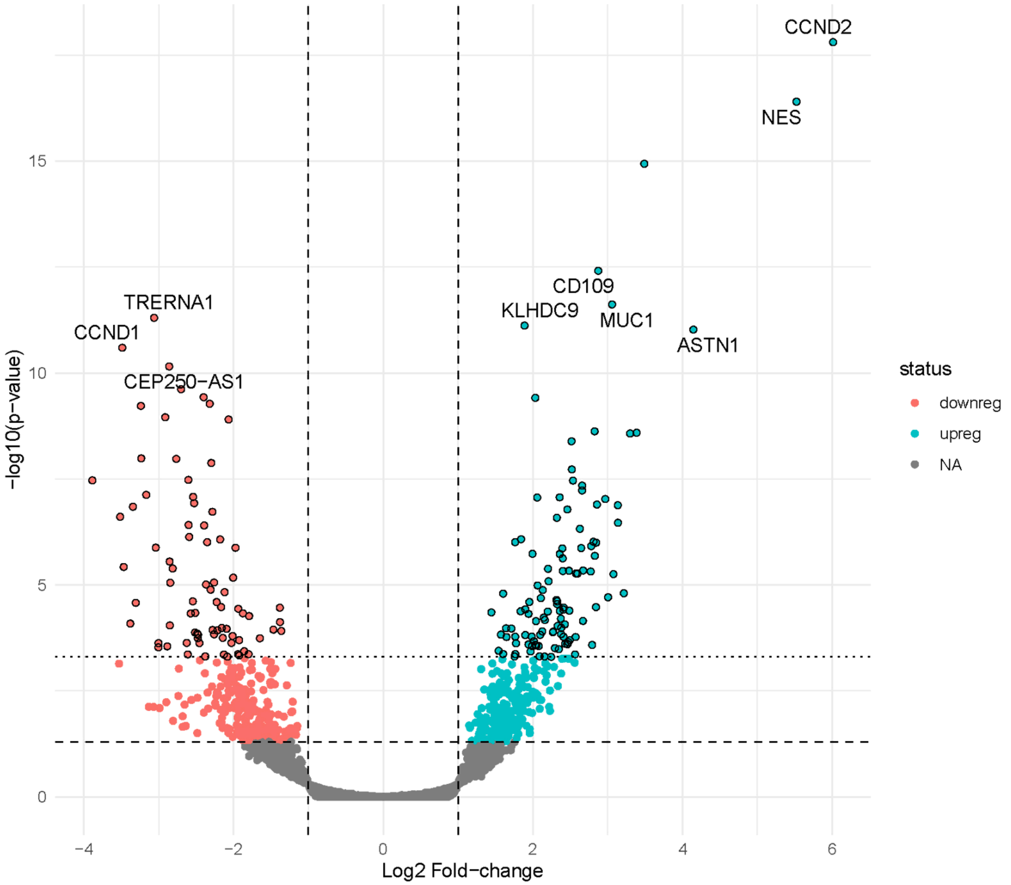

b

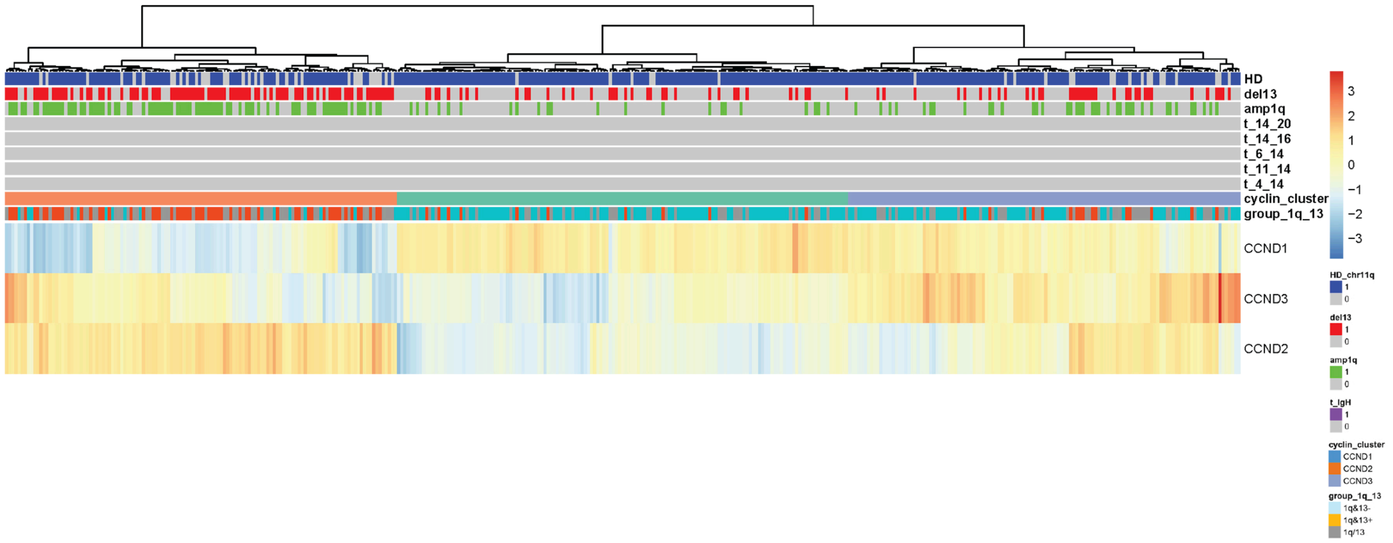

Supplementary Figure S3: (a) Volcano plot of significantly expressed genes among 1q&13+ and 1q&13- patients, excluding from the analysis patients carrying t-IgH (n=397 independent samples). (b) Heatmap describing the unsupervised clustering of 3 CCND genes. Columns represent patients. Colors are scaled row-wise according to the normalized expression values of genes. The 1q&13 classification is plotted over the heatmap (orange = 1q&13+, blue= 1q&13-, green = 1q/13+); the analysis excludes patients carrying t-IgH.

Supplementary Figure S4

BO dataset

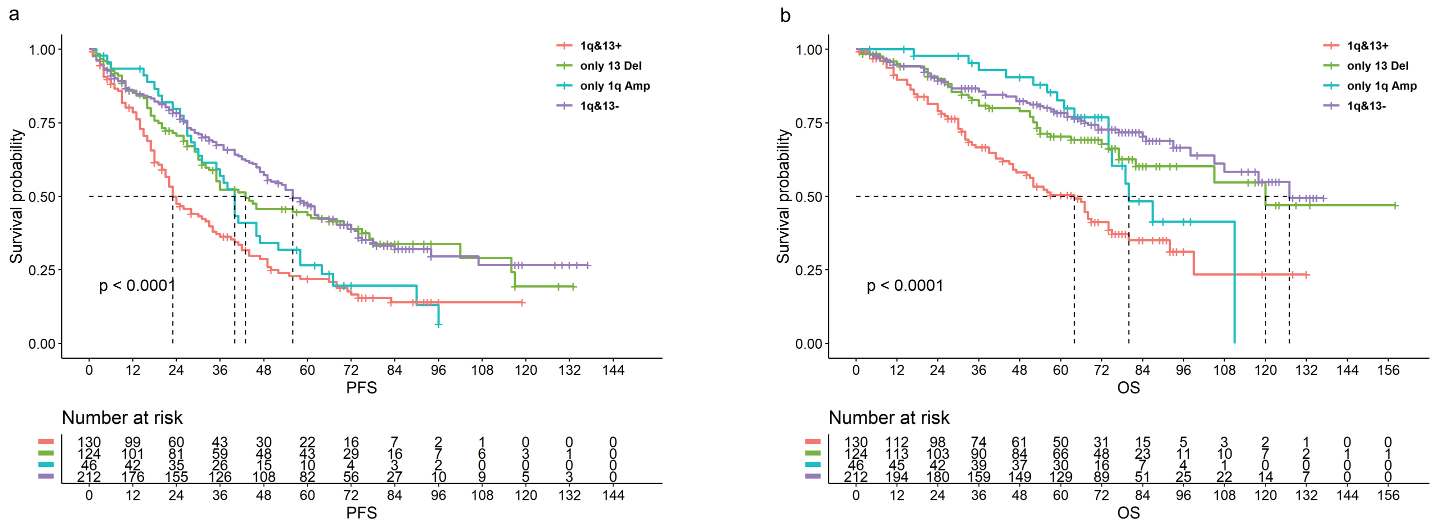

CoMMpass dataset

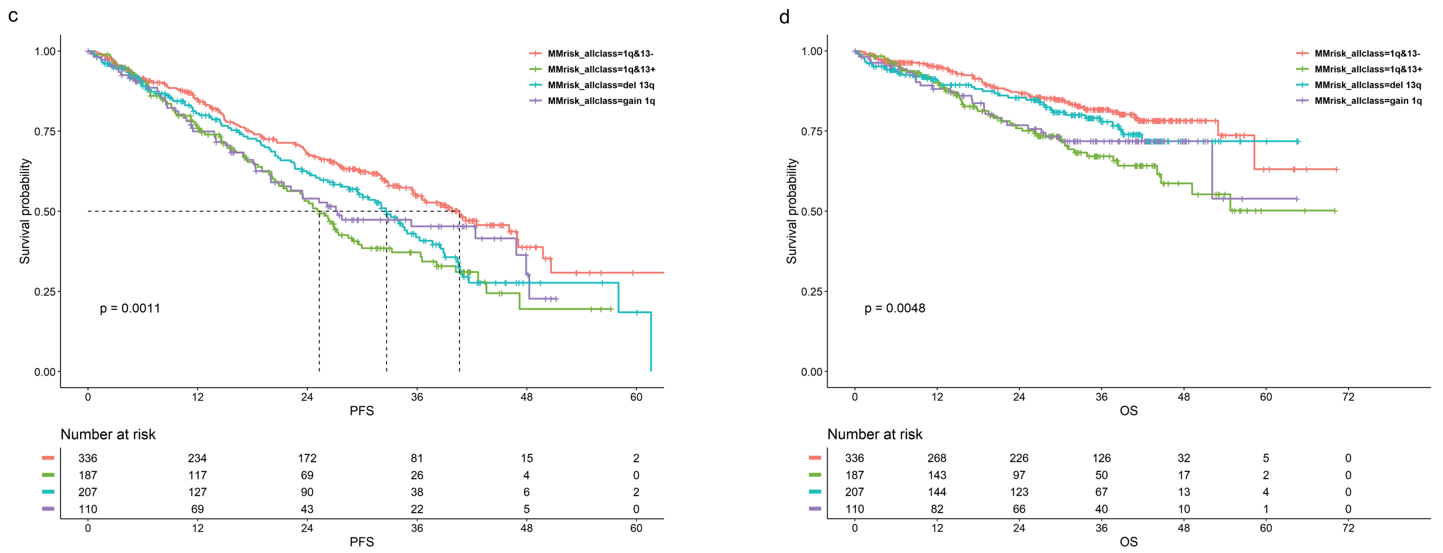

Supplementary Figure S4: Clinical impact of 1q&13 classification. a, b) Effect on PFS and OS of 1q&13 classification, including patients carrying either gain1q or loss13q alone, BO dataset (n=512 patients). c, d) Effect on PFS and OS of 1q&13 classification, including patients carrying either gain1q or loss13q alone, CoMMpass dataset (n=752 patients). Log-rank tests were used to compute reported p-values.

Supplementary Figure S5

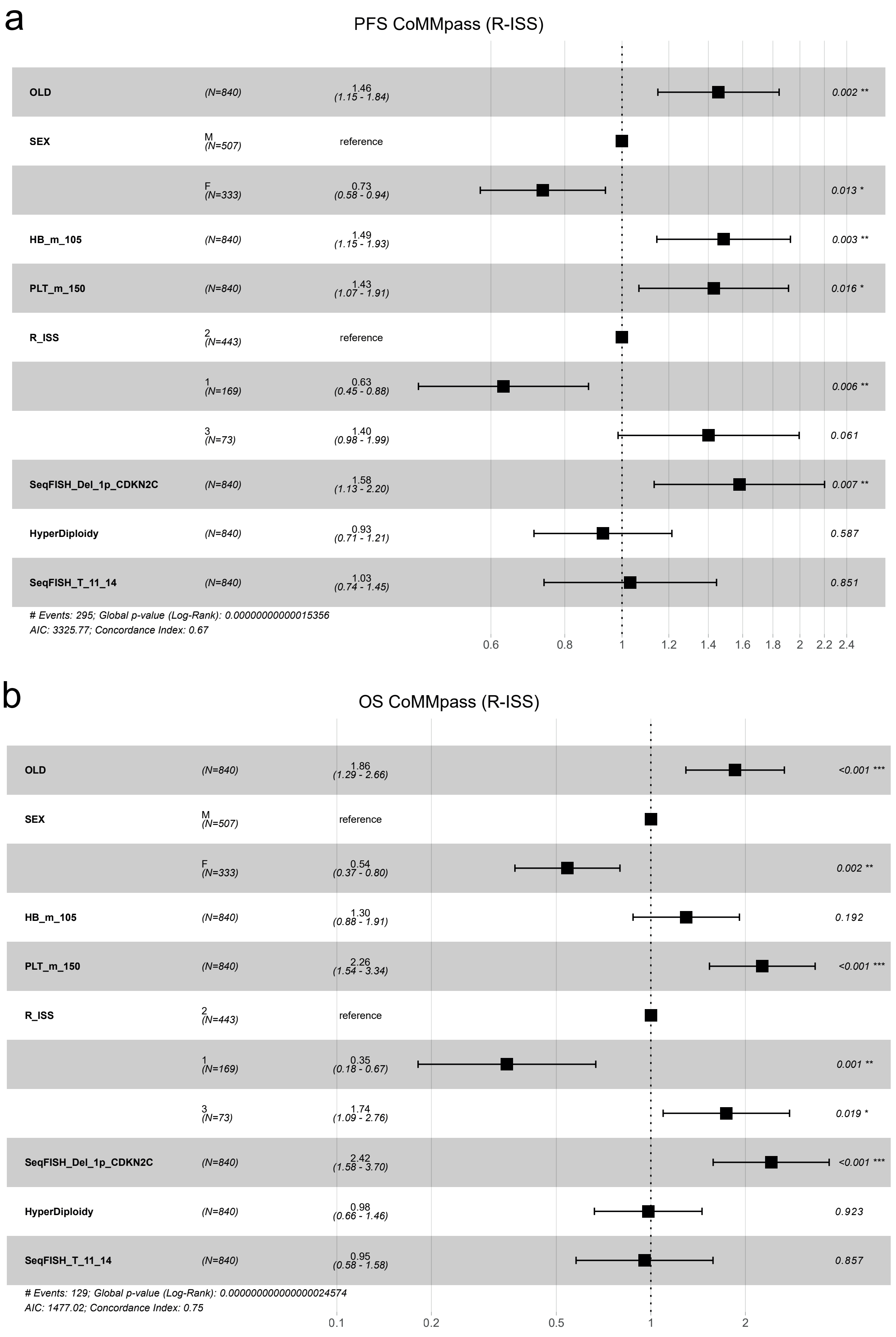

Supplementary Figure S5: Forest plots of Cox multivariate analyses to explore R-ISS Hazard Ratios (HR) in CoMMpass dataset. a) PFS model, b) OS model. The squares along the center line represent point estimates of the HR of variables included in the models, while horizontal lines extending from the squares represent 95% confidence intervals for each variable's HR.

Supplementary Table S1

a

	Overall	ISS 1	ISS 2	ISS 3	non-classified	p	test
n	840	296	287	224	33		
MMrisk_class (%)						0.060	
1q&13-	336 (40.0)	135 (45.6)	109 (38.0)	79 (35.3)	13 (39.4)		
1q&13+	187 (22.3)	56 (18.9)	69 (24.0)	59 (26.3)	3 (9.1)		
1q/13	317 (37.7)	105 (35.5)	109 (38.0)	86 (38.4)	17 (51.5)		
MMrisk_class_t_CCND2 (%)						0.002	
1q&13-	336 (40.0)	135 (45.6)	109 (38.0)	79 (35.3)	13 (39.4)		
1q&13+_pure	112 (13.3)	37 (12.5)	42 (14.6)	32 (14.3)	1 (3.0)		
del 13q only	207 (24.6)	79 (26.7)	73 (25.4)	49 (21.9)	6 (18.2)		
gain 1q only	110 (13.1)	26 (8.8)	36 (12.5)	37 (16.5)	11 (33.3)		
t&1q&13+	75 (8.9)	19 (6.4)	27 (9.4)	27 (12.1)	2 (6.1)		

b

	Overall	R-ISS 1	R-ISS 2	R-ISS 3	non-classified	p	test
n	840	169	443	73	155		
MMrisk_class (%)						<0.001	
1q&13-	336 (40.0)	92 (54.4)	164 (37.0)	18 (24.7)	62 (40.0)		
1q&13+	187 (22.3)	22 (13.0)	106 (23.9)	29 (39.7)	30 (19.4)		
1q/13	317 (37.7)	55 (32.5)	173 (39.1)	26 (35.6)	63 (40.6)		
MMrisk_class_t_CCND2 (%)						<0.001	
1q&13-	336 (40.0)	92 (54.4)	164 (37.0)	18 (24.7)	62 (40.0)		
1q&13+_pure	112 (13.3)	22 (13.0)	62 (14.0)	9 (12.3)	19 (12.3)		
del 13q only	207 (24.6)	37 (21.9)	113 (25.5)	17 (23.3)	40 (25.8)		
gain 1q only	110 (13.1)	18 (10.7)	60 (13.5)	9 (12.3)	23 (14.8)		
t&1q&13+	75 (8.9)	0 (0.0)	44 (9.9)	20 (27.4)	11 (7.1)		

Supplementary Table S1: description of both “1q&13” and “t&1q&13” classifications, as distributed in standard clinical MM stratification systems: a) Multiple Myeloma International Staging System (ISS). b) Multiple Myeloma Revised International Staging System (R-ISS). Percentages are reported column-wise across the tables. Two-sided Fisher exact test was used to compute the reported pvalues.
