# Supplementary material for: Multi-dimensional scaling techniques unveiled gain1q&loss13q co-occurrence in Multiple Myeloma patients with specific genomic, transcriptional and adverse clinical features
Source: Nat Commun. 2024 Feb 20;15:1551. doi: 10.1038/s41467-024-45000-z (PMC10879136; doi:10.1038/s41467-024-45000-z)
Supplement: Supplementary file 3 — Description of Additional Supplementary Files [file 41467_2024_45000_MOESM3_ESM.pdf]

Description of Additional Supplementary Files

Supplementary Data 1: Overall CNAs ranking, according to their recurrence in the analyzed cohorts of patients; first and second Excel sheets refer to MM-BO and CoMMpass dataset, respectively.

Supplementary Data 2: Clinical and genomic variables of patients stratified according to the 1q&13 classification (MM-BO dataset). For each risk class, two Excel sheets are present, the first one for numerical and the second for categorical variables. For categorical variables, two-sided Fisher's Exact test p-values were computed, and for continuous variables, two-sided Wilcoxon-Mann-Whitney test p-values were computed.

Supplementary Data 3: Clinical and genomic variables of patients stratified according to the 1q&13 classification (CoMMpass dataset). For each risk class, two Excel sheets are present, the first one for numerical and the second for categorical variables For categorical variables, two-sided Fisher's Exact test p-values were computed, and for continuous variables, two-sided Wilcoxon-Mann-Whitney test p-values were computed.

Supplementary Data 4: Number of significantly deregulated genes (DEGs) between pairwise 1q&13 classification groups.

Supplementary Data 5: Results of Gene Sets Enrichment Analysis (GSEA): list of differentially affected pathways (FDR<0.25 and p-value<0.5). First and second sheets refer to down-regulated and up-regulated pathways, respectively. Nominal p-values ("NOM p-val" column) to assess the statistical significance of the enrichment scores ("ES" column) were computed using default GSEA permutation tests. FDR q-values ("FDR q-val" column) were computed after adjusting for gene set size and multiple hypotheses testing by using the default GSEA distribution ratio approach.

Supplementary Data 6: Significantly deregulated genes (DEGs) between pairwise 1q&13 classification groups, with abs(Fold Change) >1. P-values were computed by using Bayes-moderated t-tests (using the "treat" function of "limma" R-package).

Supplementary Data 7: Baseline clinical treatment-related features of patients stratified according to 1q&13 classification (CoMMpass dataset). For categorical variables, two-sided Fisher's Exact test p-values were computed, and for continuous variables, two-sided Wilcoxon-Mann-Whitney test pvalues were computed.
