# Supplementary material for: Multi-dimensional scaling techniques unveiled gain1q&loss13q co-occurrence in Multiple Myeloma patients with specific genomic, transcriptional and adverse clinical features
Source: Nat Commun. 2024 Feb 20;15:1551. doi: 10.1038/s41467-024-45000-z (PMC10879136; doi:10.1038/s41467-024-45000-z)
Supplement: Supplementary file 11 — Reporting Summary [file 41467_2024_45000_MOESM11_ESM.pdf]

nature portfolio

Corresponding author(s): Carolina Terragna

Last updated by author(s): Dec 6, 2023

Reporting Summary

Nature Portfolio wishes to improve the reproducibility of the work that we publish. This form provides structure for consistency and transparency in reporting. For further information on Nature Portfolio policies, see our Editorial Policies and the Editorial Policy Checklist.

Statistics

For all statistical analyses, confirm that the following items are present in the figure legend, table legend, main text, or Methods section.

n/a	Confirmed	
☐	☒	The exact sample size (*n*) for each experimental group/condition, given as a discrete number and unit of measurement
☒	☐	A statement on whether measurements were taken from distinct samples or whether the same sample was measured repeatedly
☐	☒	The statistical test(s) used AND whether they are one- or two-sided<br>*Only common tests should be described solely by name; describe more complex techniques in the Methods section.*
☐	☒	A description of all covariates tested
☐	☒	A description of any assumptions or corrections, such as tests of normality and adjustment for multiple comparisons
☐	☒	A full description of the statistical parameters including central tendency (e.g. means) or other basic estimates (e.g. regression coefficient) AND variation (e.g. standard deviation) or associated estimates of uncertainty (e.g. confidence intervals)
☐	☒	For null hypothesis testing, the test statistic (e.g. *F*, *t*, *r*) with confidence intervals, effect sizes, degrees of freedom and *P* value noted<br>*Give P values as exact values whenever suitable.*
☒	☐	For Bayesian analysis, information on the choice of priors and Markov chain Monte Carlo settings
☒	☐	For hierarchical and complex designs, identification of the appropriate level for tests and full reporting of outcomes
☐	☒	Estimates of effect sizes (e.g. Cohen's *d*, Pearson's *r*), indicating how they were calculated

Our web collection on statistics for biologists contains articles on many of the points above.

Software and code

Policy information about availability of computer code

Data collection	No software was used for data collection
Data analysis	GitHub repository: https://github.com/andrea-poletti-unibo/1q-13_paper. DOI: https://doi.org/10.5281/zenodo.10277460.<br>Chromosome Analysis Suite (ChAS) v3.3,<br>GSEA version 4.1.0 software,<br>R programming language, version 4.2,<br>R packages: RawCopy v1.1, ASCAT 2.5.2, GISTIC 2.0, stat 4.3.1, ggvenn 0.1.10, ggExtra 0.10.1, ggpubr 0.6.0, GenomicRanges 1.52.0, corrplot 0.92, factoextra 1.0.7, rgl 1.2.1, limma 3.56.2, glimma 2.10.0, edgeR 3.42.4, Vegan 2.6-4, survival 3.5-5, survminer 0.4.9

For manuscripts utilizing custom algorithms or software that are central to the research but not yet described in published literature, software must be made available to editors and reviewers. We strongly encourage code deposition in a community repository (e.g. GitHub). See the Nature Portfolio guidelines for submitting code & software for further information.

Data

Policy information about availability of data

All manuscripts must include a data availability statement. This statement should provide the following information, where applicable:

- Accession codes, unique identifiers, or web links for publicly available datasets
- A description of any restrictions on data availability
- For clinical datasets or third party data, please ensure that the statement adheres to our policy

All the patients included in clinical trials have been de-identified and only baseline clinical variables were used in the paper analyses.
As per Editor's approval, all the raw data (SEG files, Copy Number Profiles and clinical data) are openly accessible at the following GitHub repository: https://github.com/andrea-poletti-unibo/1q-13_paper - DOI: https://doi.org/10.5281/zenodo.10277460.
Data from the "CoMMpass" study are available from dbGAP under the accession code phs000748.v1.p1.

Research involving human participants, their data, or biological material

Policy information about studies with human participants or human data. See also policy information about sex, gender (identity/presentation), and sexual orientation and race, ethnicity and racism.

Reporting on sex and gender	No gender-based analysis was carried out as this covariate is not known to impact the biology of Multiple Myeloma and data was not collected.<br><br>On the contrary, sex and age data were collected and described in table1 and table2. Accordingly, these covariates were considered in the clinical analyses (univariate and multivariate survival models).
Reporting on race, ethnicity, or other socially relevant groupings	No race, ethnicity, or other socially relevant groupings analysis was carried out as this covariate is not known to impact the biology of Multiple Myeloma and data was not collected.
Population characteristics	All the covariates relative to the population included in this study are listed and described in Table 2
Recruitment	n=513 patients were either previously enrolled in the EMN02, or in BO2005 clinical trias, or consecutively treated in our Institution in the context of the daily clinical practice, in order to avoid any possible selection bias.
Ethics oversight	The study was approved by Area Vasta Emilia Centro ethics review board (17/2015/U/Tess and 149/2018/Sper/AOUBo) and complied with all relevant ethical regulations.

Note that full information on the approval of the study protocol must also be provided in the manuscript.

Field-specific reporting

Please select the one below that is the best fit for your research. If you are not sure, read the appropriate sections before making your selection.

☒ Life sciences ☐ Behavioural & social sciences ☐ Ecological, evolutionary & environmental sciences

For a reference copy of the document with all sections, see nature.com/documents/nr-reporting-summary-flat.pdf

Life sciences study design

All studies must disclose on these points even when the disclosure is negative.

Sample size	N= 513 samples in Bologna dataset (1 per patient). All patients' samples for which both clinical and genomic (SNP array) data were available at time of analysis were included in this study.
Data exclusions	No data were excluded
Replication	All the experimental findings are completelely reproducible since they are based on bioinformatic analyses.
Randomization	No randomization nor allocation was needed to perform this retrospective observational study.
Blinding	No group allocation was performed, consequently blinding is not applicable in this study.

Reporting for specific materials, systems and methods

We require information from authors about some types of materials, experimental systems and methods used in many studies. Here, indicate whether each material, system or method listed is relevant to your study. If you are not sure if a list item applies to your research, read the appropriate section before selecting a response.

Materials & experimental systems

n/a	Involved in the study
☒	☐ Antibodies
☒	☐ Eukaryotic cell lines
☒	☐ Palaeontology and archaeology
☒	☐ Animals and other organisms
☐	☒ Clinical data
☒	☐ Dual use research of concern
☒	☐ Plants

Methods

n/a	Involved in the study
☒	☐ ChIP-seq
☒	☐ Flow cytometry
☒	☐ MRI-based neuroimaging

Clinical data

Policy information about clinical studies
All manuscripts should comply with the ICMJE guidelines for publication of clinical research and a completed CONSORT checklist must be included with all submissions.

Clinical trial registration	This study is not an interventional clinical trial.
Study protocol	This study is not an interventional clinical trial.
Data collection	These information have been published, as described in "Patients" section
Outcomes	This study is not an interventional clinical trial.
